# Globally ncRNAs Expression Profiling of TNBC and Screening of Functional lncRNA

**DOI:** 10.3389/fbioe.2020.523127

**Published:** 2021-01-21

**Authors:** Aman Chandra Kaushik, Aamir Mehmood, Xiangeng Wang, Dong-Qing Wei, Xiaofeng Dai

**Affiliations:** ^1^Wuxi School of Medicine, Jiangnan University, Wuxi, China; ^2^School of Life Sciences and Biotechnology, Shanghai Jiao Tong University, Shanghai, China

**Keywords:** triple-negative breast cancer, long non-coding RNA, machine learning, lncRNA, mRNA, miRNA

## Abstract

One of the most well-known cancer subtypes worldwide is triple-negative breast cancer (TNBC) which has reduced prediction due to its antagonistic biotic actions and target’s deficiency for the treatment. The current work aims to discover the countenance outlines and possible roles of lncRNAs in the TNBC via computational approaches. Long non-coding RNAs (lncRNAs) exert profound biological functions and are widely applied as prognostic features in cancer. We aim to identify a prognostic lncRNA signature for the TNBC. First, samples were filtered out with inadequate tumor purity and retrieved the lncRNA expression data stored in the TANRIC catalog. TNBC sufferers were divided into two prognostic classes which were dependent on their survival time (shorter or longer than 3 years). Random forest was utilized to select lncRNA features based on the lncRNAs differential expression between shorter and longer groups. The Stochastic gradient boosting method was used to construct the predictive model. As a whole, 353 lncRNAs were differentially transcribed amongst the shorter and longer groups. Using the recursive feature elimination, two lncRNAs were further selected. Trained by stochastic gradient boosting, we reached the highest accuracy of 69.69% and area under the curve of 0.6475. Our findings showed that the two-lncRNA signs can be proved as potential biomarkers for the prognostic grouping of TNBC’s sufferers. Many lncRNAs remained dysregulated in TNBC, while most of them are likely play a role in cancer biology. Some of these lncRNAs were linked to TNBC’s prediction, which makes them likely to be promising biomarkers.

## Introduction

Breast cancer (BC) is the most common form of cancer and one of the main reasons for cancer deaths among women around the globe (Torre et al., 2015). Triple-negative breast cancer (TNBC) is a subclass of breast-related cancers, identified as having low prediction due to its destructive biotic actions and lack of molecular-based targets for the treatment (Bianchini et al., 2016). As this cancer arises from various histological origins, it shows significant genomic and clinical heterogeneity both in the inter and intra affiliated individuals (Polyak, 2011). It can be demarcated via the absenteeism of estrogen and progesterone receptors (ER and PR) countenance and unavailability of the receptor for epidermal growth factor 2 for humans (HER2) amplification (Bosch et al., 2010).

This type of BC contributes to only 16% of the total diagnosis reports and is widely accepted as a notable aggressive subtype of BC. There exist very limited approaches for the treatment of TNBC due to the absence of significant beneficial marks. Therefore, it becomes essential to discover novel techniques for targets it has been a struggle to improve the treatment of TNBC. Recent break-throughs of sequencing techniques have demonstrated that expressive genses in human genomes are only around 2% (Alexander et al., 2010). The remainder of the human genome is majorly transcripted into ncRNA molecules (Carninci et al., 2005). Among all the ncRNAs, the long non-coding RNAs (lncRNAs), which are demarcated as 200 nucleotides, have lengthy copies, and lack protein-expression functionality (Ponting et al., 2009), have attracted ever-increasing research nowadays because of their regulatory potential at transcriptional, posttranscriptional, and chromosomal levels (Kornienko et al., 2013).

Furthermore, lncRNAs are widely dysregulated in numerous cancers including BC, which makes them likely to be investigative and predictive biomarkers or suitable potential cancer targets (Malih et al., 2016; Zhang R. et al., 2016; Zhang Y. et al., 2016). Several lncRNAs have been detected to play a role in the regulation of the TNBC’s development. For instance, lncRNA *LINP1* is observed to be overexpressed, increasing the repair of double-strand DNA disruption in the TNBC. Hindering *LINP1* upsurges the BC cell response sensitiveness toward radiotherapy (Zhang Y. et al., 2016).

*LINK-A* flattens the initiation of BRK kinase, hence activating signaling of the normoxic HIF1α in TNBC, encouraging the glycolysis reprogramming and tumor development (Lin et al., 2016). Similar to miRNA, which is extensively applied as a diagnostic and prognostic biomarker in cancer (Zhang et al., 2014), lncRNA has also been identified as a biomarker in various forms of cancers (Zheng et al., 2014; Huang et al., 2015). Nevertheless, the capability of lncRNA for the prognosis of TNBC is still not completely explored. Here, we utilized the RNA-seq data from TANRIC to develop a two-lncRNA panel prognostic model. The robustness of the panel is evaluated by 10-fold cross-validation. Advancements in the genomic and RNA sequencing technologies, as well as bioinformatics approaches, have enabled us to explore additional lncRNAs. But, the information about lncRNAs and TNBC is quite limited and the countenance outline, roles and biological machinery regarding lncRNAs in TNBC need to be extensively investigated (Kapusta and Feschotte, 2014; Wang et al., 2017). Cancer has had a broad impact on society, it is important to recognize effective and robust tools that could change the impact of cancer (Kaushik and Sahi, 2017; Kaliamurthi et al., 2018; Kaushik et al., 2018, 2019a, b; Kaushik et al., 2020a, b, c, d).

Previous reports have exposed that lncRNAs are significantly involved in cancer and the countenance outline or particular mutations regarding lncRNA genes are caught up during the growth and cancer development. Thus, in the current work, we have excavated and examined data from various repositories, with the goal of pointing out signatures of lncRNAs in TNBC, forming the basis for future work and these predicted lncRNAs linked to TNBCs that are likely to be promising biomarkers.

## Materials and Methods

### Gene Expression Catalogs

The information about lncRNAs, miRNAs, mRNAs, copy number alterations (CAN), mutational expression, and gene alterations were directly retrieved from The Cancer Genome Atlas (TCGA) repository. The “TNBC (TCGA)” cancer study was chosen with “Mutation and CAN” as a preferred data type before examining the cell cycle’s genomic alterations on TNBC. As this data is obtained from publically available databases, no informed consent was required.

### Acquisition and Analysis of Genomic Variations

The available tumor samples were used to summarize the genomic modifications for cell cycle control. This involved mutations, CAN (amplifications and homozygous deletions), gyphs, and color coding for summarizing gene expression variations. This was an initial stage to comprehend various forms of TNBC’s gene signaling. Furthermore, mutual uniqueness along with co-occurrence among the cell cycle control was examined as well. Contrary to this, gene-linked activities connected with a specific type of cancer are mostly time conflicting within the cluster of tumors i.e., only a single biological ocurrence is anticipated to occur in each sample of cancer. Another situation is the occurence happening where numerous genes are altered in the same sample. This was a primary approach to folding data associated with diverse gene signaling in TNBC.

### TNBC’s Cell Cycle Control Mutations

Triple-negative breast cancers show RB1 mutations/deletions which conciliate the reliability of cell cycle control via the Rb/E2F/CDK4/6 pathway, along with numerous alterations in DNA damage response genes like BRCA1. These tumors similarly tend to be vastly aneuploid with near universal loss of the TP53 role, persistent CCNE1 DNA amplifications and PTEN loss of function. Several cellular reliance studies have confirmed that TNBC tumors are contingent on the shaft assembly checkpoint and show high expression levels of mitotic checkpoint genes like TTK, BUB1, MAD2, AURKB, and DNA repair proteins, apparently due to their genomic instability. Copy gains of CDK4 are collective across BC types, with the highest occurrence in HER2 + tumors. Moreover, pathognomonic amplification of ERBB2, mutations of TP53, PIK3CA, and PTEN and DNA amplification of CCND1 are also numerous in this subtype. Consequently, heterogeneity in mechanisms occur transversely across subtypes, the existence of alterations that contribute to aberrant progression of the cell cycle is a hallmark of TNBC cells.

The mutations cell cycle control helped in specifying the site and occurrence of all changes in the Pfam protein’s areas. The entire cell cycle’s extent is denoted by colored bars while the ratio of amino acids is represented in the gray. Protein’s domains are displayed by the red, blue, and green boxes. The lines and points signify the position and rate of genes, respectively. The nonsense or frameshift mutations are colored red, missense mutations in green, and in-frame deletions are colored in black (Fang et al., 2015).

### Co-appearance System and Hub-Genes

This study aimed to analyze differentially expressed microRNA, lncRNAs, and mRNAs to identify prognosis-related RNAs; where differentially expressed miRNAs, lncRNAs, and mRNAs between BC and normal samples were analyzed through public databases. Then differentially expressed miRNAs, lncRNAs and mRNAs between both (ER+ and ER-) samples were screened. lncRNAs were further analyzed with patient prognosis. Analyzing the co-expression data revealed several miRNA–mRNA–lncRNAs with an expressively interrelated expression. A co-expression grid of this data is built based on their association constants. The grid is a huge system having positive and negative associations with nodes and edges. Out of this, few significant hub-genes were chosen for visual analysis (Figure 3).

### Statistical Based Survival Analysis

The correlation analysis was carried out by plotting a scattered graph of lncRNAs, miRNAs, mRNAs, CAN, mutational expression, or protein level for every sample. To carry out correlation analysis, a scattered graph of lncRNAs, miRNAs, mRNAs, CAN, mutational expression, or protein applied for survival analysis, the Kaplan–Meier graphs with log-rank tests were conducted to equate the global and healthy existence of TNBC having a minimum or zero alteration within the query gene(s). The over-expressed samples were recognized by maintaining a verge of Z > 2 (mean expression over 2 SDs). A value of 0.05 was kept as standard.

### Patient Data Retrieval

The expression information regarding lncRNA was obtained from TANRIC^1^ (Li et al., 2015) (The Atlas of NcRNA In Cancer)^2^ of all BC patients recorded in TCGA^3^ (Ciriello et al., 2015). The associated clinical information was obtained from the Genomic Data Commons (GDC; Jensen et al., 2017). Patients (*n* = 180) were identified as TNBC if ER and HER2 expression were both under the corresponding median expression. Purity estimation was performed for the 180 TNBC patients using agreement transparency approximation (Aran et al., 2015) and Clonal Heterogeneity Analysis Tool (Li and Li, 2014). Patients were filtered out if and only if both purity estimators were below 60%.

### LncRNA Feature Selection

To identify promising discriminative lncRNAs prognostic of BC survival, the limma R package was employed (Ritchie et al., 2015) to identify differentially transcribed lncRNAs between patients stratified by overall survival time (with 3 years as the split point). The threshold was set to log_2_ fold change >2 and the accustomed **p**-value <0.01. Differentially expressed lncRNAs were used as the input that was subjected to random forest recursive feature elimination (RF-RFE), which ranked the features on recurrent bases in the order of importance based on the decrease of the Gini index. To establish variable importance, this study first uses the module dependency graph (MDG) method for variable selection (Strobl et al., 2007).

MDG is the amount of all reductions in Gini impurity because of a given inconstant, regularized by the number of trees (Menze et al., 2009).

The Gini index at node v of RF, Gini(P).

$$\frac{1}{n}\sum_{k=1}^{n}{\left(P_{k}-O_{k}\right)}^{2}$$

The equation signifies the likelihood of class association by P and the factual class association by O. It shadows that a lesser Brier value indicates higher correctness, and therefore the optimum presentation signified by the Brier value is equal to 0.

### Predictive Modeling

The random forests approach or random decision forests approach is an assortment learning approach that could be used for cataloging and regression. The random forest approach can be assumed to be accurate, random points out random sampling and features while forest points out multiple decision trees. A single decision tree could effect over-fitting problems, but may be focused with the cluster results of multiple decision-making trees. Hence, random forest is a unified learning approach. In the Gradient Boosting protocol, the utmost usual base learner is the decision tree. For the construction of a predictive model of survival groups, we have used a gradient boosting machine (GBM). It is an influential collaborative learning technique which has gained a great performance level in several biomedical missions. The GBM plays a significant role in lifting a set of alleged weak learners to configure an enhanced group of classifiers. GBM has been used as a controlled categorizing algorithm to allow patient stratification employing the algorithms that have been developed through recent studies. A GBM classifier comprises a group of decision hierarchies. Due to this feature, GBMs are proficient for heterogeneous data stemming, multi-variate from distinct measurement procedures and have distinct numerical gauges and allocation in the situation as ours. Every decision tree is assigned a weight in the GBMs. Several decision trees get created and weighted during the iterative training mechanism of GBMs. Predicted GBM performance is critically influenced by an optimal number of boosting steps and can, for instance, be revealed by cross-validation. Remarkably, it is not a compulsion that GBMs utilize all variables that are present in the classification information, but possibly only a few subsets. Therefore, the resultant grader is likely to be sparse. Additionally, it is feasible to obtain a degree of significance of each particular inconstant for the resulting classifier. The comparative drop of non-conformist towards the drilling information is reflected through this measure.

## Results

### Functional Characterization and Changes in the Genomic Landscape

**FIGURE 1:**
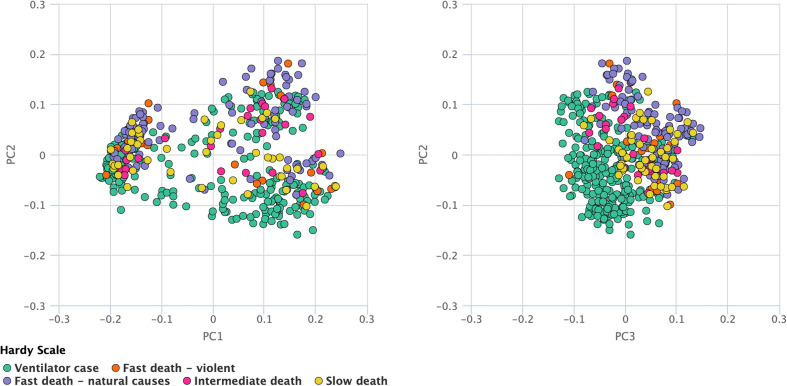
Principal component analysis. The TCGA and survival data is used for plotting the principal components. This analysis suggests that events which happened in the cell cycle control were likely to happen again in the TNBC as illustrated in Figure, where green dots depict the ventilator case, red dots depict fast death, violet dots show fast death (natural causes), pink dots represent intermediate death and yellow dots show slow deaths.

Recent research on lncRNAs suggests its involvement in many crucial cell cycle regulators like cyclins, CDKs, CDK inhibitors, pRB, and p53 and these lncRNAs play important role in epigenetic regulators, transcription factor regulators, post-transcription regulators, and protein scaffolds. Cell cycle-regulated lncRNAs control cell cycle regulators through various mechanisms and are involved in the diversity and reliability of the crucial cell cycle regulations. Most of the cases, particularly in the cell cycle control, endure alterations in which almost all of them were observed to be missense mutations. Besides this, deep deletions and some amplifications have also been incorporated. The rest of all the cases were comprised mainly of the truncating and missense mutations. The joint selectness breakdown suggests that events that happened in the cell cycle control were likely to happen again in the TNBC as illustrated in Figure 1. The stage and age of patients’ survival are shown in the supplementary data (Supplementary Figure 1).

**FIGURE 2:**
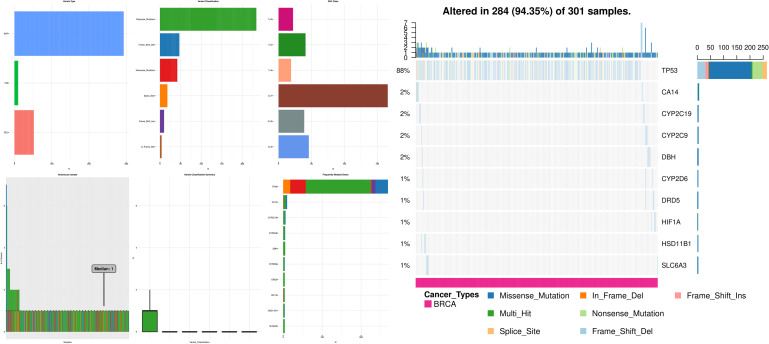
Illustrations here represent that missense mutations and truncating mutations mediate gene signaling in the case of TNBC, where the first panel depicts the variant type, the second panel depicts the variant per sample, the third panel depicts the variant classification, the fourth panel depicts the variant classification summary, the fifth panel depicts the SNV class, the sixth panel depicts the frequently mutated genes and the seventh panel depicts the CNA events occurred in the cell cycle control.

Changes were observed in most of the cases, 284 out of 301 (94.35%), where the majority of them were missense mutations. The shared exclusivity breakdown implies that CNA events that occurred in cell cycle control were liable to occur again in the TNBC as shown in Figure 2.

### Expression Landscape Among TNBC Patients Using the Two-lncRNA Predictive Model

Based on layering, 180 sufferers were separated into two groups (classes). The capability of an administered machine learning-based classifier’s capability was assessed for stratifying fresh patients into the right classes, performed through a 10× repetitive tenfold cross-validation technique. It implies that a grader was competent on a 9/10 scale of 180 premenopausal sufferers having non-threatening breast modifications and projection of the sufferer class constituted for the leftover 1/10 of the patients. We directed the ultimate machine learning classifier among all 180 patients elected biomarkers that gave rise to stratified patient classes. Thereafter, we employed this classifier onto 180 premenopausal BC patients. Six out of these 180 patients were expected to drop with the higher possibility (>90%) into any one of the group. This implies that these six BC patients show a major resemblance to 180 patients who had non-threatening breast modifications. Additionally, patients coming under cluster 2, signify a higher resemblance to the sufferers falling in this subtype. Therefore, we inferred sufferers falling into group 2 as the patients with a high risk of BC are in this sub-class.

**FIGURE 3:**
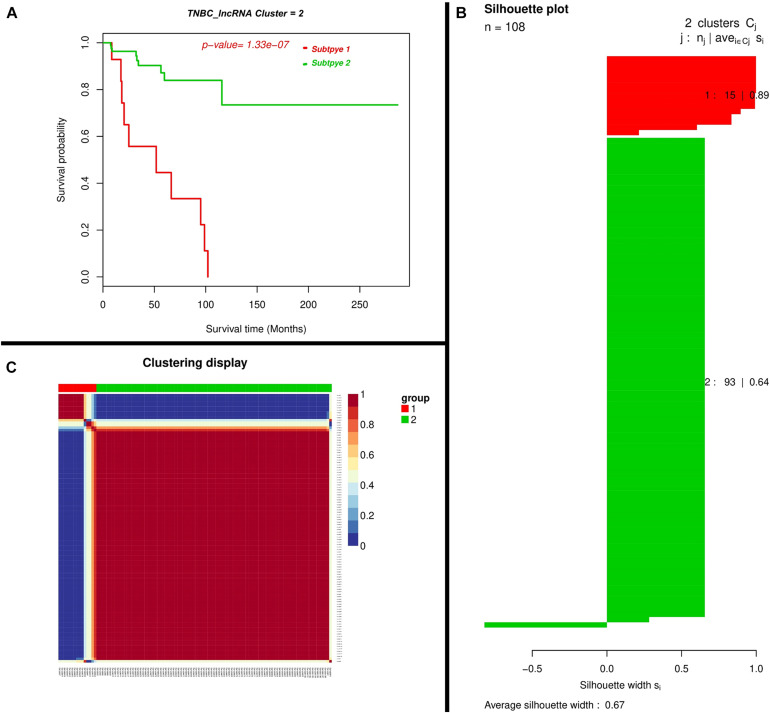
Two-lncRNA panels for TNBC prognosis. **(A)** Kaplan–Meier Plotter showing the predictive value of the lncRNA panel. **(B)** Silhouette plot; Consent medium with super-imposed dendrogram of hierarchical clustering. The consensus matrix depicts the relative frequency of two patients falling into the same cluster across repeated runs. A clear separation of two patient subgroups (red blocks) can be seen. Patients in these groups frequently fall into the same cluster and **(C)** Silhouette plot of two patient subgroups, respectively. The *x*-axis shows the cluster silhouette for each patient on the *y*-axis. The cluster silhouette is a measure of how similar each patient is compared to patients in its cluster and the closest patient from other clusters. The silhouette measure ranges from 0 to 1, where 1 indicates a perfect agreement of the assumed cluster assignment with patient distances. The average cluster silhouette for all patients in cluster 1 and 2 is shown. The overall average silhouette index was 1.

At the threshold of log2 fold change >2 and accustomed *p*-values <0.01, we found lncRNAs may exert a more profound biological impact than a single gene by the virtue of its intrinsic regulatory nature. We performed a two-lncRNA predictive model for TNBC prognosis and the Expression landscape of lncRNA between survival groups’ patients. We identified 0.67 average silhouette width and 0.000000133 *p*-values of two lncRNA clusters respectively. LncRNAs found were expressed among a silhouette plot and distributed among different biotypes (Figure 3), encompassing long non-coding survival probability and clustering of lncRNAs respectively. To explore the diagnostic potential of ncRNAs, we executed hierarchical clustering for differentially expressed lncRNAs shown in Figure 3.

### Prognostic Value of Biomarkers

Triple-negative breast cancer clustering has one important characteristic that is the assortment of a approporate biomarker which is responsible for cluster induction in patients. In the present scenario, a total of six biomarkers from lncR, miR, and mR are detected that turned into a clear differentiation of two sufferers’ groups having elevated silhouette indices (Table 1).

**TABLE 1:** The table shows the six identified biomarkers amid these grouping solutions, representing their increased constancy.

lncRNA	miRNA	mRNA
LINKA (LINC01139)	hsa-miR-21-5p	TRIM25
*BCAR4*	miRNA-29c	*CDC2*

### Survival Analysis

For the survival analysis of TNBC, Kaplan–Meier plots were used. The global survival has also been analyzed that reveals concurrent cases that are not associated with decreased survival.

### Co-expression Analysis of lncRNAs With mRNAs

**FIGURE 4:**
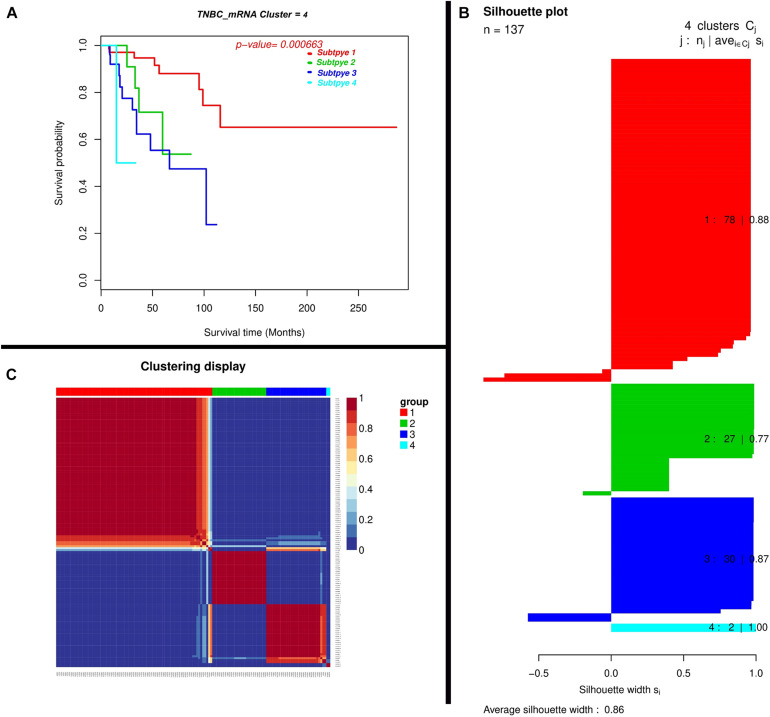
Depiction of a two-mRNA predictive model for TNBC prognosis and expression landscape of mRNA between survival groups for the prognostic grouping of TNBC patients. **(A)** Kaplan-Meier Plotter showing the predictive value of the mRNA panel. **(B)** Silhouette plot; Consensus matrix with super-imposed dendrogram of hierarchical clustering. The consensus matrix depicts the relative frequency of two patients falling into the same cluster across repeated runs. A clear separation of two patient subgroups (red blocks) can be seen. Patients in these groups frequently fall into the same cluster and **(C)** Silhouette plot of two patient subgroups, respectively. The *x*-axis shows the cluster silhouette for each patient on the *y*-axis. The cluster silhouette is a measure of how similar each patient is compared to patients in its cluster and the closest patient from other clusters. The silhouette measure ranges from 0 to 1, where 1 indicates a perfect agreement of the assumed cluster assignment with patient distances. The average cluster silhouette for all patients in cluster 1 and 2 is shown. The overall average silhouette index was 1.

We found mRNAs may exert a more profound biological impact than a single gene by the virtue of its intrinsic regulatory nature. We performed two-mRNA predictive model for the TNBC prognosis and the Expression landscape of mRNA between survival groups’ patients. We identified 0.86 average silhouette width and 0.000663 as the *p*-value of two mRNA clusters respectively. The mRNAs were found to be expressed among silhouette plots and distributed among different biotypes (Figure 4), encompassing long non-coding survival probability and clustering of the mRNA respectively. To explore the diagnostic potential of ncRNAs, we executed hierarchical clustering for differentially expressed mRNAs shown in Figure 4.

### Co-expression Analysis of lncRNAs With miRNAs

**FIGURE 5:**
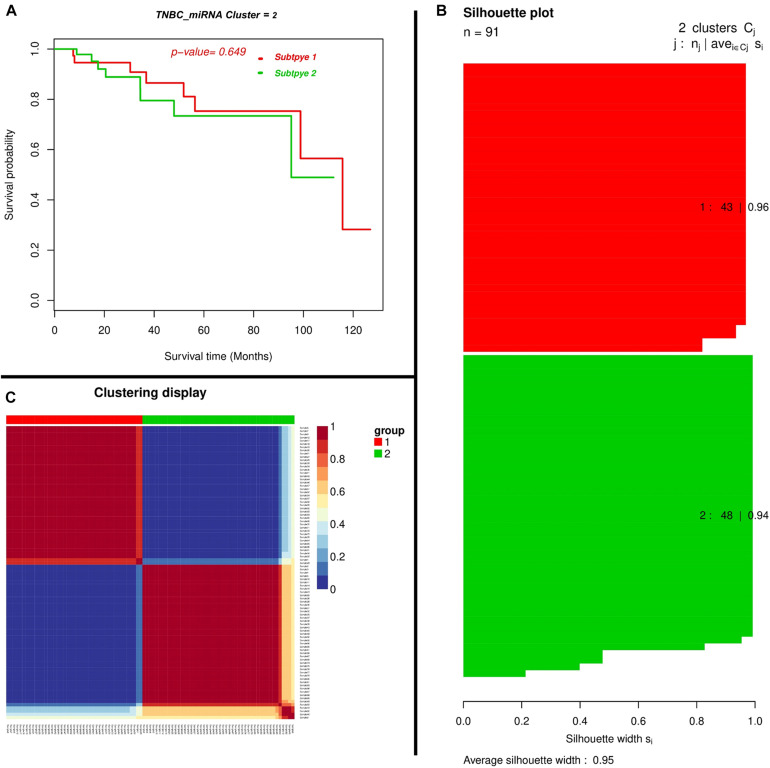
Depicting a two-miRNA predictive model for the TNBC’s prognosis and expression landscape of miRNA between survival groups for the prognostic grouping of TNBC patients. **(A)** Kaplan–Meier Plotter showing the predictive value of the miRNA panel. **(B)** Silhouette plot; Consensus matrix with super-imposed dendrogram of hierarchical clustering. The consensus matrix depicts the relative frequency of two patients falling into the same cluster across the repeated runs. A clear separation of two patient subgroups (red blocks) can be seen. Patients in these groups frequently fall into the same cluster, and **(C)** Silhouette plot of two patient subgroups, respectively. The *x*-axis shows the cluster silhouette for each patient on the *y*-axis. The cluster silhouette is a measure of how similar each patient is as compared to patients in its cluster and the closest patient from other clusters. The silhouette measure ranges from 0 to 1, where 1 indicates a perfect agreement of the assumed cluster assignment with patient distances. The average cluster silhouette for all patients in cluster 1 and 2 is shown. The overall average silhouette index was 1.

We found miRNAs may exert a more profound biological impact than a single gene by the virtue of its intrinsic regulatory nature. We performed a two-miRNA predictive model for the TNBC prognosis and the Expression landscape of the miRNA between survival groups’ patients. We identified 0.95 average silhouettes’ width and 0.649 as a *p*-value of the two miRNA clusters, respectively. The miRNAs were found to be expressed among silhouette plots and distributed among different biotypes (Figure 5), encompassing long non-coding survival probability and clustering of miRNA, respectively. To explore the diagnostic potential of ncRNAs, we executed hierarchical clustering for differentially expressed miRNAs shown in Figure 5.

### Benefits of the Proposed Diagnostic Approach

A multi-omics method has been used as the base for the diagnostic approach shown here and the objective was to construct the model based on biomarkers which satisfies two conditions:

1. They are corresponding to one another about the genetic functions associated with each individual, which can be accredited to tumor advancement and progression by differential patterns.
2. It is probable that systematic and disease-specific biomarker patterns are presented by them which can specifically be of immense medical utility by making risk evaluation modalities based on blood tests associated with tumor development in females susceptible to TNBC before the medical confirmation of the disease.

### Biological Construal of the Chosen Biomarkers

We evaluated the correlative impact of all of the six biomarkers which were discovered, having the capability to place the inmates amid the increased and lowered-risk classes by the customized machine learning-based grader. To confirm if the corresponding biomarkers upsurge or decline the comparative possibility of a sufferer to be placed in the group with increased risk.

## Discussion

As mentioned earlier, the administration of the premenopausal TNBC (preTNBC) is difficult, because of escalating pervasiveness, no peculiar screening programs, less developed targeted prevention and predictive diagnostics. PubMed statistics reflect that this area attracts more attention which represents a constant increase in an annual number of papers dedicated to the field beginning with a single paper in the year 1971 extending to 4341 PubMed-registered papers in the year 2019. Nevertheless, in total, there are at present merely 4341 and 8 papers that can be noted as exclusively devoted to the TNBC hazard evaluation and TNBC forecast, correspondingly. It is a shockingly low volume of published reports signifying apparent shortfall in the research activities associated with the field. Extensive-cohort reports persist to convey on level-1 family history regarding TNBC, the tremendously or heterogeneously intense breast tissue, overweight/obesity, anthropometric parameters, abnormal alcohol consumption, decreased physical activity, disease-predisposing reproductive history, and history of benign breast biopsy as the vital danger issues for both before and after the menopausal TNBC, having several additional consequences for each other, subject to the cause and population.

With the use of multi-omic information methodology, the present plan disclosed two evident and strong disjoint classes with high against low TNBC resemblance in premenopausal, TNBC exempt cases. These classes were influenced by an extremely consistent subgroup of merely six biomarkers. Additionally, we designed a machine learning mockup, which is capable of foretelling any pre-menopausal female as a fellow of low or increased TNBC hazard cluster based on the conventional biomarker panel.

As emphasized, the stated low and elevated TNBC risk subgroups falling in premenopausal patients free from the TNBC need extra attestation through large-scale patient analyses. We think our current outcomes as revitalizing; even though, the reproducibility count is presently hard to assess. Carrying our biomarker board along escorted by the advanced layering algorithm into the medical training will need a medical test. These types of analyses would need validation that the projected highly dangered persons are comparatively frequent to the illness on TNBC throughout their lifespan in comparison to individuals with minimum danger based on the assessment organization. It is after the percentage of risk throughout life in the classes is observed to be relatable to the cost of layering, to make an absolute judgment regarding the medical use of the intended approach. Hence, the represented work must be taken into consideration as one of the first encouraging moves to benefit possibly affected inmates, wellbeing and humanity at large.

## Conclusion

Our findings provide a new vision for exploring biological functions of ncRNAs in the TNBC, and screening novel potential biomarkers through two-ncRNAs sign might be a biomarker for the prognostic grouping of TNBC sufferers.

## Possible Future Research Directions of ncRNAs in TNBC

1. Possible future research directions of ncRNAs in TNBC should be converging on the personalized approach, which will help further develop our knowledge ofeach kind of epigenetic mediator such as panobinostat, vorinostat, and entinostat. Moreover, by isolating potential treatment biomarkers (lncRNAs), which are occupied in epigenetic machineries through the enlistment of chromatin modification complexes.
2. Clinicians in the near future should be competent to provide tailored treatments in concurrence to the lncRNA stratification or explicit lncRNA expression outlines in different patient sub-groups in TNBC.
3. Conversely, utmost lncRNAs prompt in low level in precise tissue and RNA deprivation may occurr in paraffin-bedded tissue, as a result the probe-combinations are simply mis-estimated. Some probe-combinations may be prohibited from the stereochemical structure of RNA. To reconnoiter this problem, forthcoming studies evaluating a huge number of tissues are still required.

## Data Availability Statement

The datasets generated for this study are available on request to the corresponding author.

## Author Contributions

AK designed the experiment, performed the entire computational experiments, assisted in writing the manuscript, analyzed the data, and wrote the manuscript. All authors read the manuscript, advised on method development, and have approved the final version of the manuscript.

## Conflict of Interest

The authors declare that the research was conducted in the absence of any commercial or financial relationships that could be construed as a potential conflict of interest.

## Footnotes

1 https://bioinformatics.mdanderson.org/public-software/tanric/

2 https://ibl.mdanderson.org/tanric/_design/basic/index.html

3 https://www.cancer.gov/about-nci/organization/ccg/research/structural-genomics/tcga
