# Supplementary material for: Globally ncRNAs Expression Profiling of TNBC and Screening of Functional lncRNA
Source: Front Bioeng Biotechnol. 2021 Jan 21;8:523127. doi: 10.3389/fbioe.2020.523127 (PMC7860147; doi:10.3389/fbioe.2020.523127)
Supplement: Supplementary file 1 [file Data_Sheet_1.docx]

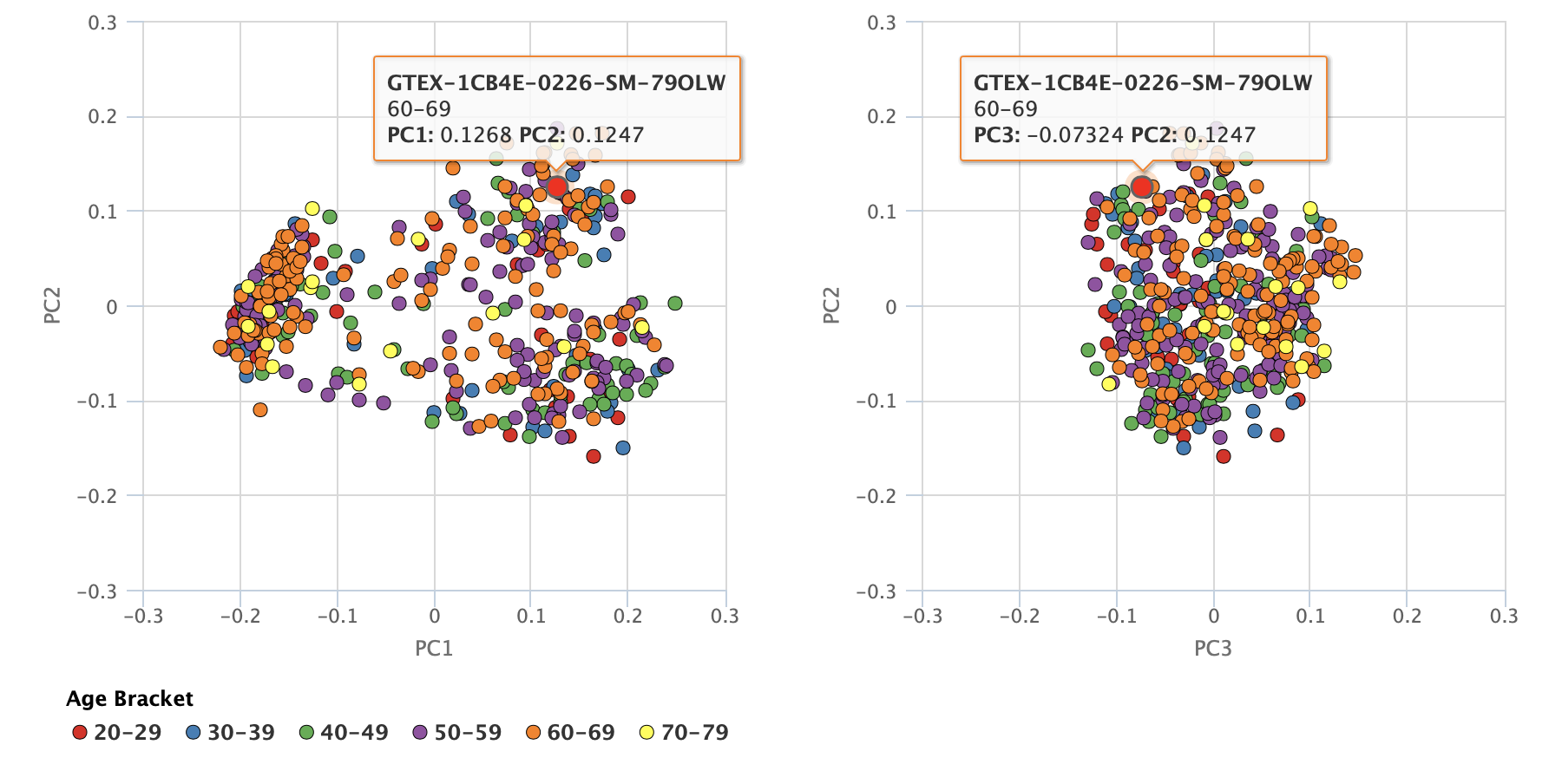


**Fig. S1.** Principal component analysis. The GTEX and survival data is used for plotting the principal components. This analysis suggests that events happened in the cell cycle control were responsible to happen again in TNBC as illustrated in Figure S1, where red dots depicts that 20-29 patient, blue dots depicts that 30-39 patient, green dots depicts that 40-49 patient, violet dots depicts that 50-59 patient, orange dots depicts that 60-69 patient and yellow dots depicts that 70-79 patient.

**2.3 TNBC’s cell cycle control mutations**

TNBCs show RB1 mutations/deletions which conciliation the reliability of cell cycle control via the Rb/E2F/ CDK4/6 pathway, along with numerous alterations in DNA damage response genes like BRCA1. These tumors similarly tend to be vastly aneuploid with near universal loss of TP53 role, persistent CCNE1 DNA amplifications and PTEN loss of function. Several cellular reliance studies have confirmed that TNBC tumors are contingent on the shaft assembly checkpoint and show high expression levels of mitotic checkpoint genes like TTK, BUB1, MAD2, AURKB and DNA repair proteins, apparently due to their genomic instability. Copy gains of CDK4 are collective across breast cancer types, with the highest occurrence in HER2+ tumors. Moreover, pathognomonic amplification of ERBB2, mutations of TP53, PIK3CA, and PTEN and DNA amplification of CCND1 are also numerous in this subtype. Consequently, heterogeneity in mechanisms occur transversely subtypes, the existence of alterations that contribute to aberrant progression of the cell cycle is a hallmark of TNBC cells.
